# Effects of Low Dose Metformin on Metabolic Traits in Clozapine-Treated Schizophrenia Patients: An Exploratory Twelve-Week Randomized, Double-Blind, Placebo-Controlled Study

**DOI:** 10.1371/journal.pone.0168347

**Published:** 2016-12-14

**Authors:** Chih-Chiang Chiu, Mong-Liang Lu, Ming-Chyi Huang, Po-Yu Chen, Yen-Kuang Lin, Shih-Ku Lin, Chun-Hsin Chen

**Affiliations:** 1 Department of Psychiatry, Taipei City Psychiatric Center, Taipei City Hospital, Taipei, Taiwan; 2 Department of Psychiatry, School of Medicine, College of Medicine, Taipei Medical University, Taipei, Taiwan; 3 Department of Psychiatry, Wan Fang Hospital, Taipei Medical University, Taipei, Taiwan; 4 Graduate Institute of Medical Science, School of Medicine, Taipei Medical University, Taipei, Taiwan; 5 Biostatistics Center, Taipei Medical University, Taipei, Taiwan; Heidelberg University, GERMANY

## Abstract

**Background:**

Metformin has been used for alleviating metabolic abnormalities in patients with schizophrenia. The lowest dose of metformin to treat metabolic abnormalities in clozapine-treated patients is 1000 mg/d. This study was designed to determine whether metformin at 500 mg/d and 1000 mg/d is effective in improving the metabolic profiles of clozapine-treated patients with pre-existing metabolic abnormalities, and whether its effectiveness depends on metformin dosage.

**Methods:**

In this 12-week, randomized, double-blind, placebo-controlled trial, metformin at 500 mg/d or 1000 mg/d was prescribed to clozapine-treated patients with schizophrenia who had pre-existing metabolic abnormalities. The recruited patients underwent physical and laboratory evaluations at weeks 4, 8, and 12. The outcomes were any changes in metabolic traits.

**Results:**

Among the 96 clozapine-treated patients with schizophrenia screened for the trial, 55 patients with pre-existing metabolic abnormalities were randomly assigned to placebo (n = 18), metformin dosage at 500 mg/d (n = 18), and metformin dosage at 1000 mg/d (n = 19) groups. The body weight (BW) of patients in the metformin 1000 mg/d group significantly decreased, by a mean of 0.97 kg over the 12 week trial period. Moreover, patients in the metformin at 500 mg/d and 1000 mg/d groups had a significant decrease in body mass index (BMI) after 12 weeks, with the mean decrease being 0.70 and 0.50 kg/m^2^, respectively. No significant changes were observed in the other metabolic parameters of patients in the three groups.

**Conclusions:**

Our results demonstrated that a low metformin dosage of either 500 mg/d or 1000 mg/d for 12 weeks slightly reduced the BW and BMI of clozapine-treated patients with pre-existing metabolic abnormalities. A longer period of treatment with a larger sample is warranted to determine the factors that influence the metformin treatment response.

**Trial Registration:**

ClinicalTrials.gov NCT02751307

## Introduction

Body weight (BW) gain and metabolic abnormalities are major side effects observed in antipsychotic-treated patients with schizophrenia. Clozapine, the most effective agent for treatment-resistant schizophrenia [1,2], has the highest propensity for inducing BW gain and metabolic abnormalities among all the available antipsychotics [3,4]. In our previous survey, 38% of patients receiving clozapine or olanzapine had metabolic syndrome [5]. In the CATIE phase-3 study, patients receiving clozapine had an increased BW and higher blood levels of glucose, triglyceride (TG), and glycosylated hemoglobin [6]. Therefore, it is crucial to control metabolic abnormalities in clozapine-treated patients with schizophrenia.

Metformin, prescribed for patients with non-insulin-dependent diabetes for controlling blood glucose levels, has been reported to induce BW loss in various insulin-resistant patient groups [7]. Within psychiatry, metformin has been suggested to be the most effective medication for attenuating antipsychotic-related BW gain and metabolic abnormalities [8,9]. Three studies have evaluated the effect of metformin on metabolic profiles in clozapine-treated patients with schizophrenia. Treatment with metformin at a dosages of 1000 mg/d for 14 weeks [10], 1000 mg/d for 20 weeks [11], or 1500 mg/d for 24 weeks [12] effectively reduced BW or BMI and improved metabolic profiles without obvious side effects when it was taken by clozapine-treated patients with schizophrenia. However, the BW of metformin-treated patients may return to the higher baseline after discontinuation of metformin for at least 24 weeks [12]. Long-term use of metformin may be necessary for patients with schizophrenia at a high risk of metabolic syndrome. Therefore, selecting an optimal metformin dosage for patients with metabolic abnormalities is critical. The lowest dosage of metformin used in previous studies for treating metabolic abnormalities in clozapine-treated patients is 1000 mg/d [10,11]. In the present study, we compared the effects of a placebo, metformin at 500 mg/d, and metformin at 1000 mg/d on metabolic profiles in clozapine-treated patients with metabolic abnormalities. We hypothesized that metformin at 500 mg/d and 1000 mg/d would have a beneficial effect on metabolic profiles compared with the placebo, and that a small metformin dosage of 500 mg/d may have equal effect to the larger dose of 1000 mg/d.

## Methods

In this 12-week, randomized, double-blind, placebo-controlled trial, a placebo, metformin 500 mg/d, or metformin 1000 mg/d was prescribed to clozapine-treated patients with schizophrenia that had pre-existing metabolic abnormalities. The study was approved by an institutional review board and was conducted at Taipei Medical University–Wan Fang Hospital and Taipei City Psychiatric Center from May 1^st^, 2013 to January 31^st^, 2015. All clinical investigation was conducted according to the principles expressed in the Declaration of Helsinki. We firstly screened clozapine-treated patients and then enrolled eligible patients in this clinical trial. Written informed consent was obtained from all patients before the screening. Psychiatrists who referred the patients to this study were responsible for the evaluation of the patients’ competence to provide informed consent. The evaluation of competence was dependent on clinical judgment without using any criteria. For incompetent patients, we explained the study to their caregivers and obtained informed consent from them. We registered the trial at ClinicalTrials.gov (identifier: NCT02751307). Although the trial was not registered until after it was completed because of administrative negligence, we followed CONSORT guidelines for a randomized trial (S1 Table).

### Patients in the screening phase

Patients diagnosed with schizophrenia or schizoaffective disorder according to the *Diagnostic and Statistical Manual of Mental Disorders*, Fourth Edition; aged 20–65 years; and who had taken clozapine for at least 3 months were invited to the screening. Clinical interviews were conducted to confirm the diagnosis, and medical records were evaluated for collecting patient demographics and clinical information, namely diagnosis, age of onset, and clozapine dosage prescribed. We used the Positive and Negative Syndrome Scale (PANSS) [13] to evaluate the patients’ psychopathology.

### Measurements

The height, BW, waist circumference (WC), and blood pressure (BP) of all patients were measured. The body mass index (BMI) was calculated as the BW in kilograms divided by the height squared in meters. After overnight fasting, blood was collected for analysis of fasting plasma glucose (FPG), TG, and high-density lipoprotein cholesterol (HDL-C) levels. Fasting TG, FPG, and HDL-C levels were measured using an automated system under the guidance of the manufacturer (Roche Cobas C501).

### Patients in the metformin trial

Patients who underwent the screening were enrolled in the present trial if they had at least one of the following metabolic abnormalities: BMI ≥ 24; WC > 90 cm (men) or 80 cm (women); fasting serum TG level ≥ 150 mg/dL; fasting serum HDL-C level ≤ 40 mg/dL (men) or 50 mg/dL (women); systolic BP ≥ 130 or diastolic BP ≥ 85 mm Hg; current use of antihypertensive agents; or FPG level = 100–126 mg/dL. The exclusion criteria were the following: history of diabetes mellitus (DM); current use of hypoglycemic or hypolipidemic agents; pregnancy; allergy to metformin; a creatinine level > 1.4 ng/dL; an abnormal liver function test result; or chronic cardiopulmonary insufficiency.

### Trial procedures

Patients recruited in the trial were randomized, in a 1:1:1 ratio, to treatment with a placebo, metformin 500 mg/d, or metformin 1000 mg/d by a computer-generated allocation sequence with blocks of six. The randomization was conducted by a research assistant, who was blinded to the patient’s status. To ensure the concealment of the randomization, the metformin and placebo were provided in coded containers. The placebo was identical in appearance to the metformin tablets. All patients, caregivers, and investigators were masked to the randomization.

In the first week, 500 mg of metformin was administered in the morning to the metformin 500 mg/d and 1000 mg/d groups, and the placebo was administered to the placebo group. In the second week, the dosage was revised to 500 mg of metformin in the morning and the placebo in the evening for the metformin 500 mg/d group, 500 mg of metformin twice a day for the metformin 1000 mg/d group, and placebo twice daily for the placebo group. The clozapine dosage taken by all patients remained unchanged throughout the intervention. The recruited patients underwent physical and laboratory evaluations at weeks 4, 8, and 12.

### Statistical analyses

We used descriptive statistics to summarize the baseline clinical characteristics of the patients, analysis of variance to examine differences in the numerical variables, and the chi-square test to analyze differences in categorical variables among all groups. Additionally, we used a paired *t* test to evaluate changes in PANSS score before and after the intervention in each group, and analysis of variance to evaluate changes in PANSS scores among the three groups. Patients who received the intervention for at least four consecutive weeks were included in the analyses, which were performed according to the intention-to-treat principle. We adopted the last observation carried forward (LOCF) approach for replacing missing data, which assumes no change in missing metabolic indices after a dropout event. Furthermore, to investigate whether longitudinal survey variables changed over time, we first used the paired *t* test to determine differences between the baseline and follow-up measures. Repeated measure analyses were conducted using 2-way within-subjects analysis of variance for the examining group (df = 2, between groups), time (df = 3, within-subjects), and interaction (df = 6, interaction between time and groups) effects on the changes in metabolic profiles over time. The analyses were conducted using SPSS 20.0 (IBM, Armonk, NY). Statistical significance was indicated by p < 0.05.

### Sample size estimation

Our previous study reported that the mean BW decrease was 2.2 kg after 16-week and 3.2 kg after 24-week of 1500 mg/d of metformin intervention [12]. Another study also revealed a mean 3.2 kg BW decrease over a 12-week 750 mg/d metformin treatment [14]. Therefore, we set the difference of BW change as 2 kg in metformin group (either 500 or 1000 mg/d) and 0 kg in the placebo group. The standard deviation was set as 2 kg according to the results of our previous trial [12]. With a power of 80% and a type I error of 5%, the minimal size for each group was 16 subjects.

## Results

**Fig 1:**
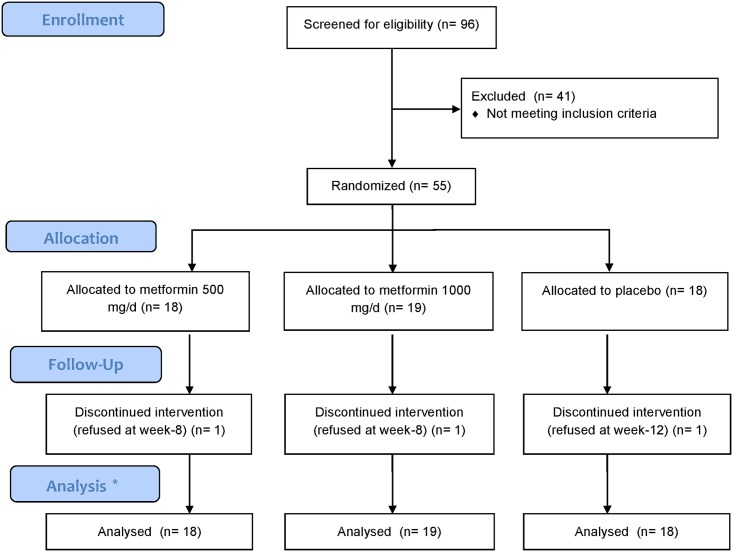
Flowchart of recruitment process—We adopted the LOCF approach. Patients who continued the intervention for at least 4 weeks were included in the analyses.

Fig 1 is a flowchart of the whole recruitment process. We screened 96 clozapine-treated patients with schizophrenia. Table 1 presents the patient demographics, clinical information, and metabolic profiles at the baseline. Among all the patients, 55 fulfilled the criteria for the clinical trial and were randomly assigned to either the metformin 500 mg/d (n = 18), metformin 1000 mg/d (n = 19), or placebo (n = 18) groups. Table 2 lists the demographic data, clinical information, and metabolic profiles of the patients in each group. The patients in the metformin 1000 mg/d group were significantly older than those in the other groups, and those in the metformin 500 mg/d group had a significantly lower average BW and a lower BMI and WC. No other significant differences were discovered among the three groups.

**Table 1:** Demographic, clinical characteristics, and baseline metabolic profiles in all screened patients.

	N = 96
	Mean (SD) or N (%)
Age, year	44.9 (8.8)
Gender, male/female	48(50) / 48(50)
Current Smoker	19 (19.8)
Current alcohol drinker	2 (2.1)
Onset of age, year	21.7 (5.8)
Duration of illness, year	23.3 (8.7)
Clozapine dose, mg/day	269.6 (100.8)
BW, kg	67.8 (11.5)
BMI, kg/M^2^	25.4 (3.7)
BMI ≥ 24	55 (57.3)
Waist, cm	90.8 (9.8)
Systolic BP, mmHg	121.7 (13.3)
Diastolic BP, mmHg	77.3 (10.2)
Triglyceride, mg/dL	132.2 (83.2)
HDL-C, mg/dL	48.7 (13.8)
FPG, mg/dL	104.9 (20.7)

Abbreviation: BMI, body mass index; BP, blood pressure; BW, body weight; FPG, fasting plasma glucose; HDL-C, high-density lipoprotein cholesterol.

**Table 2:** Baseline characteristics of patients in placebo, metformin at 500 mg/d, and metformin at 1000 mg/d groups.

	Placebo	Met 500 mg/d	Met 1000 mg/d	p value
	(n = 18)	(n = 18)	(n = 19)	
Age, year	44.2 (10.0)	42.1 (8.4)	**50.3 (6.1)**	0.01
Gender, male/female	8/10	8/10	8/11	0.98
Current smoker	3 (21.4)	5 (27.8)	3 (18.8)	0.81
Current alcohol drinker	1 (6.7)	1 (5.6)	0	0.60
Onset of age, year	20.8 (6.5)	21.7 (5.9)	20.8 (5.6)	0.68
Duration of illness, year	25.1 (9.7)	21.4 (8.5)	27.8 (6.0)	0.08
Clozapine dose, mg/day	261.5 (119.3)	284.8 (101.5)	263.5 (67.4)	0.79
BW, kg	71.1 (10.4)	**62.8 (9.7)**	69.7 (9.4)	0.03
BMI, kg/M^2^	26.7 (3.8)	24.6 (3.5)	26.6 (3.2)	0.15
BMI ≥ 24	15 (83.3)	9 (50.0)	14 (73.2)	0.04
Waist, cm	94.4 (8.2)	88.7 (9.3)	93.6 (8.6)	0.11
Systolic BP, mmHg	121.3 (14.0)	121.0 (10.2)	122.5 (13.0)	0.93
Diastolic, BP, mmHg	78.9 (14.1)	76.7 (6.8)	76.2 (10.9)	0.73
Triglyceride, mg/dL	148.1 (82.0)	127.1 (99.9)	131.4 (73.8)	0.74
HDL-C, mg/dL	44.9 (12.4)	51.7 (12.9)	53.9 (15.1)	0.08
FPG, mg/dL	98.9 (10.9)	104.4 (10.6)	106.4 (11.7)	0.11

Abbreviation: BMI, body mass index; BP, blood pressure; BW, body weight; FPG, fasting plasma glucose; HDL-C, high-density lipoprotein cholesterol; Met, metformin.

Three patients discontinued the study before its completion: one in the metformin 500 mg/d group in week 8, one in the metformin 1000 mg/d group in week 8, and one in the placebo group in week 12. Table 3 presents the metabolic profile changes during the 12-week study using LOCF data. The BW of patients in the metformin 1000 mg/d group had significantly decreased over the 12 week trial. The metformin 500 mg/d and 1000 mg/d groups had decreased BMI at week 12, and the metformin 500 mg/d group further had a transiently decreased FPG at weeks 4 and 8. No significant changes were observed in the other metabolic parameters for patients in any group. The interaction between time and group was significant in glucose but not in any other metabolic parameters.

**Table 3:** Changes of metabolic traits among patients in placebo, metformin at 500 mg/d, and metformin at 1000 mg/d groups.

	Baseline	Week-4	Week-8	Week-12	Time effect	Group effect	Time*group
	Mean (SD)						
*BW*, *kg*					df = 3, F = 3.51, p = 0.02	df = 2, F = 3.73, p = 0.03	df = 6, F = 0.73, p = 0.62
*Placebo*	71.1 (10.4)	71.2 (10.7)	70.6 (10.5)	71.0 (10.8)			
*Met 500 mg/d*	62.8 (9.7)	62.6 (10.3)	62.4 (10.2)	62.1 (10.7)			
*Met 1000 mg/d*	69.7 (9.4)	69.7 (9.3)	69.2 (8.9)	68.7 (8.9)*			
*BMI*, *kg/M*^*2*^					df = 3, F = 8.28, p < 0.01	df = 2, F = 2.25, p = 0.12	df = 6, F = 0.73, p = 0.63
*Placebo*	26.7 (3.8)	26.6 (3.8)	26.4 (3.7)	26.4 (3.7)			
*Met 500 mg/d*	24.6 (3.4)	24.5 (3.5)	24.4(3.6)	23.9 (3.6)*			
*Met 1000 mg/d*	26.6 (3.2)	26.5 (3.2)	26.3 (3.1)	26.1 (3.0)*			
*Waist*, *cm*					df = 3, F = 3.24, p = 0.02	df = 2, F = 2.12, p = 0.13	df = 6, F = 0.91, p = 0.49
*Placebo*	94.4 (8.2)	93.2 (7.9)	93.6 (8.1)	93.3 (8.5)			
*Met 500 mg/d*	88.7 (9.3)	88.9 (9.7)	87.5 (9.6)	87.5 (10.0)			
*Met 1000 mg/d*	93.6 (8.6)	93.8 (9.1)	93.2 (8.6)	91.4 (10.6)			
*Systolic BP*, *mmHg*					df = 3, F = 0.63, p = 0.60	df = 2, F = 0.75, p = 0.48	df = 6, F = 0.51, p = 0.80
*Placebo*	120.2 (13.5)	121.1 (14.8)	121.4 (14.5)	124.4 (15.6)			
*Met 500 mg/d*	121.0 (10.2)	117.2 (13.0)	116.8 (14.4)	117.2 (10.2)			
*Met 1000 mg/d*	122.5 (13.0)	119.4 (15.9)	121.6 (13.6)	122.2 (16.6)			
*Diastolic BP*, *mmHg*					df = 3, F = 0.45, p = 0.72	df = 2, F = 0.33, p = 0.72	df = 6, F = 0.24, p = 0.96
*Placebo*	78.9 (14.1)	80.0 (12.5)	76.9 (10.0)	79.0 (12.9)			
*Met 500 mg/d*	76.7 (6.8)	78.1 (10.8)	76.3 (10.9)	78.3 (11.0)			
*Met 1000 mg/d*	76.2 (10.9)	75.9 (10.1)	76.5 (10.2)	76.7 (10.6)			
*Triglyceride*, *mg/dL*					df = 3, F = 0.13, p = 0.94	df = 2, F = 0.49, p = 0.62	df = 6, F = 0.98, p = 0.44
*Placebo*	148.1 (82.0)	134.8 (49.0)	132.5 (52.0)	145.3 (66.8)			
*Met 500 mg/d*	127.1 (99.9)	116.3 (65.2)	121.4 (70.1)	123.5 (76.1)			
*Met 1000 mg/d*	131.4 (73.8)	145.6 (80.2)	151.3 (92.6)	140.6 (70.4)			
*HDL-C*, *mg/dL*					df = 3, F = 1.47, p = 0.23	df = 2, F = 2.82, p = 0.07	df = 6, F = 0.34, p = 0.96
*Placebo*	43.9 (12.4)	42.8 (11.2)	42.1 (10.6)	43.0 (10.7)			
*Met 500 mg/d*	51.7 (12.9)	48.7 (13.2)	49.1 (10.7)	48.3 (10.1)			
*Met 1000 mg/d*	53.9 (15.1)	52.9 (19.5)	53.7 (17.2)	52.6 (19.2)			
*FPG*, *mg/dL*					df = 3, F = 0.53, p = 0.67	df = 2, F = 2.85, p = 0.07	df = 6, F = 2.49, p = 0.03
*Placebo*	98.8 (10.9)	101.7 (11.4)	100.5 (9.1)	100.7 (15.0)			
*Met 500 mg/d*	104.4 (10.6)	98.9 (10.5)*	99.7 (9.2)**	103.4 (7.5)			
*Met 1000 mg/d*	106.4 (11.7)	108.5 (16.9)	114.8 (28.9)	105.1 (13.5)			

* p < 0.05 compared to baseline;

** p = 0.051 compared to baseline

Abbreviation: BMI, body mass index; BP, blood pressure; BW, body weight; FPG, fasting plasma glucose; HDL-C, high-density lipoprotein cholesterol; Met, metformin.

Once we had accounted for age and baseline BMI, all of the aforementioned findings were no longer significant. However, after analysis of the data of the overweight and obese patients (BMI ≥ 24), the effect of time since study initialization on BW and BMI was significant, whereas the group effect was not. Post hoc analyses demonstrated that the effect of metformin treatment on BW was noticeable at week 12 in the metformin 1000 mg/d group (p = 0.005), but not earlier or in other groups. A decrease in BMI was noted at week 12 in the metformin 500 mg/d group (p = 0.088), and a significant decrease in BMI was discovered at week 12 in the metformin 1000 mg/d group (p = 0.011). No other significant metabolic changes were noted.

Regarding safety, the mean PANSS scores at the baseline and at week 12 were 60.0 and 53.9 (p < 0.001), 53.0 and 48.6 (p = 0.01), and 54.2 and 51.4 (p = 0.04) in the placebo, metformin 500 mg/d, and metformin 1000 mg/d groups, respectively. The changes in the PANSS scores between the baseline and week 12 were not significantly different among the three groups (F = 1.48, p = 0.24). No serious adverse effects were reported. Moreover, 3, 4, and 4 patients reported nausea or vomiting, and 5, 2, and 5 patients reported diarrhea in the placebo, metformin 500 mg/d, and metformin 1000 mg/d groups, respectively. These side effects were not significantly different among the three groups.

## Discussion

This study was the first to compare the effects of different metformin dosages on the metabolic profiles of clozapine-treated patients with schizophrenia who had pre-existing metabolic abnormalities. Our results revealed that the patients in the metformin 1000 mg/d group had significantly decreased BW after 12 weeks of intervention, with a mean BW decrease of 0.97 kg (p = 0.017). Carrizo et al. reported that metformin 1000 mg/d treatment yielded a mean BW decrease of 0.87 kg and 1.87 kg after 7 and 14 weeks, respectively [10]. In our previous study, we discovered that the mean BW decrease in clozapine-treated patients that were administered 1500 mg/d of metformin was 1.3, 2.1, and 3.2 kg at weeks 8, 16, and 24, respectively. Moreover, a significant BW loss, defined as more than 7% of the BW, was observed in 3.6%, 10.7%, and 28.6% of patients at weeks 8, 16, and 24, respectively [12]. This evidence suggests that the effect of metformin treatment on BW is positively correlated with the treatment duration; however, we could not determine at what point during the treatment the minimum BW was achieved. In the Diabetes Prevention Program Outcomes study, the effect of metformin on BW was most apparent after 1 year of metformin treatment [15]. To date, the longest metformin trials in the field of psychiatry were 24 weeks [12] and 6 months [16]. Therefore, it would be ideal to study the effect of one or more years’ metformin treatment on metabolic profiles.

Treatment with 500 mg/d of metformin was not discovered to have a significant effect on the BW of those who received it. One possible explanation for this is that the metformin 500 mg/d group had a lower mean BW (62.8 kg) than the metformin 1000 mg/d group (69.7 kg) and placebo (71.1 kg) groups. However, further analysis for the overweight and obese patients in each group revealed a significant effect on BW at week 12 in the metformin 1000 mg/d group, but not in the other groups. This suggests that 500 mg daily may be too low to have an effect.

The BMI of patients in both the metformin 500 mg/d and metformin 1000 mg/d groups was significantly decreased at week 12, with a mean decrease of 0.70 kg/M^2^ (p = 0.021) and 0.50 kg/M^2^ (p = 0.017), respectively. Although metformin 500 mg/d treatment did not significantly reduce BW, it did significantly reduce the BMI at week 12. Compared with BW, the BMI may be a more suitable indicator of metabolic syndrome. WC and BMI are the most accurate surrogate markers of visceral adiposity and are strong indicators of insulin resistance [17]. Therefore, our result was compatible with that of another study examining patients treated with various antipsychotics, which reported that administering metformin at 750 mg/d for 12 weeks significantly reduced BMI [14]. As for its effect on BW, the effect of metformin treatment on BMI was not significant until 12 weeks.

Our study revealed that metformin treatment did not have a significant effect on WC. Although one recent study demonstrated that metformin at 1000 mg/d could reduce WC [11], two other studies, with treatment of metformin at either 1500 mg/d for 24 weeks [12] or 1000 mg/d for 12 weeks [10], did not find a difference in patients’ WC. Metformin treatment was, however, found to significantly reduce WC in clozapine-treated patients in a meta-analysis study [18]. The sample size in each study may have been too small and thus their power insufficient for detecting the effect of metformin treatment on WC.

Although metformin has been extensively used for treating patients with DM [19], we discovered that only metformin at a dose of 500 mg/d, and neither metformin at 1000 mg/d or the placebo, reduced FPG after 4 and 8 weeks of treatment. Furthermore, any reduction in FPG that had been made was found to be reversed at week 12. Therefore, the transient reduction in FPG at weeks 4 and 8 in the metformin 500 mg/d group may have been an artifact rather than a true change. The effect of metformin on FPG in patients with schizophrenia remains controversial. In our previous study, metformin treatment at 1500 mg/d effectively reduced FPG in clozapine-treated patients with metabolic abnormalities [12], and the effect appeared as early as 2 weeks after the start of treatment. Wu et al. discovered that metformin treatment at 750 mg/d effectively reduced FPG level within 4 weeks in patients with antipsychotic-induced BW gain [14]. However, other studies concluded that metformin treatment at 1000 mg/d had no effect on FPG levels, but did reduce insulin levels [10,16]. The different characteristics of the recruited patients and individual variation in response to metformin treatment may account for the disparities among studies. Most of the recruited patients in the current study did not have hyperglycemia, so the effect of metformin on FPG level may have been negligible.

We discovered that metformin treatment at neither 500 mg/d nor 1000 mg/d significantly affected TG levels during the 12-week intervention. In patients with psychiatric disorders, the effect of metformin on TG level remains controversial. One recent meta-analysis revealed that metformin significantly reduced serum TG levels in clozapine-treated patients, compared with a placebo [18]. However, the meta-analysis did not discuss the effect of dosage. For example, metformin at 1500 mg/d significantly reduced serum TG levels in Chen’s study [12], but metformin at 1000 mg/d did not in Carrizo’s study [10]. Moreover, metformin at 2000 mg/d significantly reduced the serum TG level in patients with BMI ≥ 27 [20]. A meta-analysis of patients with type 2 DM showed a significant effect of metformin on TG levels [21]; however, no significant effect was observed in patients administered a metformin dosage lower than 1700 mg/d. Therefore, the lower dosages (500 mg/d and 1000 mg/d) of metformin administered in our study might explain the negative findings regarding serum TG changes.

We observed no significant effect of metformin treatment at 500 mg/d and 1000 mg/d on HDL-C levels, which was in agreement with the results of a meta-analysis on clozapine-treated patients with schizophrenia [18]. Neither meta-analyses of studies involving patients with psychiatric disorders [8,22] nor type 2 DM [21] reported a significant effect of metformin on serum HDL-C levels. Therefore, further study is required to determine whether metformin affects serum HDL-C levels in patients with schizophrenia.

Our study revealed no effect of metformin treatment on BP, in agreement with the finding that metformin had no effect on BP in clozapine-treated patients with schizophrenia in one meta-analysis [18].

All recruited patients exhibited a significant improvement in their PANSS score after the 12-week intervention, but the improvement did not differ among the three groups. Metformin has previously been shown to have no effect on psychiatric symptoms in antipsychotic-treated patients [23] or clozapine-treated patients [12]. The patients in the current study had used clozapine for more than three months before being enrolled in the study, but the exact duration of clozapine use was unknown. Because the clinical efficacy of clozapine may become evident after more than three months of use [24], the improvement in PANSS scores may have been due to the effect of clozapine.

The present study had several limitations. First, the sample size was small. In estimating the sample size, we set the standard deviation of BW at 2 kg, which may have been an underestimation. This may have caused the sample size to be smaller than that required to detect differences among groups; the results were temporary and require validation in a large sample. Second, we did not evaluate the diet and physical activity of the enrolled patients. Therefore, we cannot confirm that any decrease in BW or BMI was not due to an interaction between the metformin treatment and any lifestyle changes. Third, we did not measure the levels of some metabolism-related hormones, such as insulin and leptin, which are crucial in studying changes in metabolic profiles. Fourth, the 12-week intervention was relatively short. The beneficial effect on BW was only seen at week 12. In our previous trial, the effect of metformin on BW was clearly observed after an intervention of 16–24 weeks [12]. Further study is required for determining whether a longer treatment of low-dosage metformin would have a more beneficial effect on patient’s metabolic profiles. Finally, because our trial enrolled patients taking clozapine who had one or more metabolic abnormalities but with an average BMI only bordering on overweight (24.6–26.7), the results may not be generalizable to a wider group who already have metabolic syndrome. The results, however, do suggest that early intervention with low doses of metformin had a beneficial effect on BMI in those with few metabolic abnormalities.

In conclusion, a 12-week treatment of metformin at either 500 mg/d or 1000 mg/d can lead to a slight decrease in the BW or BMI of clozapine-treated patients with schizophrenia that have pre-existing metabolic abnormalities. Longer-term treatment, larger sample sizes, and exploration of factors that influence metformin treatment response are warranted in future studies.

## Supporting Information

S1 File Approved protocol. (PDF)

S1 Table CONSORT checklist. CONSORT 2010 checklist of information to include when reporting a randomised trial.
(DOCX)
