# Correction: Effects of Low Dose Metformin on Metabolic Traits in Clozapine-Treated Schizophrenia Patients: An Exploratory Twelve-Week Randomized, Double-Blind, Placebo-Controlled Study

**DOI:** 10.1371/journal.pone.0193315

**Published:** 2018-02-16

**Authors:** Chih-Chiang Chiu, Mong-Liang Lu, Ming-Chyi Huang, Po-Yu Chen, Yen-Kuang Lin, Shih-Ku Lin, Chun-Hsin Chen

There are errors in the author affiliations. The affiliations should appear as shown here:

Chih-Chiang Chiu^1,2^, Mong-Liang Lu^2,3^, Ming-Chyi Huang^1,2^, Po-Yu Chen^1,4^, Yen-Kuang Lin^5^, Shih-Ku Lin^1,2^, Chun-Hsin Chen^2,3^

**1** Department of Psychiatry, Taipei City Psychiatric Center, Taipei City Hospital, Taipei, Taiwan, **2** Department of Psychiatry, School of Medicine, College of Medicine, Taipei Medical University, Taipei, Taiwan, **3** Department of Psychiatry, Wan Fang Hospital, Taipei Medical University, Taipei, Taiwan, **4** Graduate Institute of Medical Science, School of Medicine, Taipei Medical University, Taipei, Taiwan, **5** Biostatistics Center, Taipei Medical University, Taipei, Taiwan
