# Supplementary material for: Effects of Low Dose Metformin on Metabolic Traits in Clozapine-Treated Schizophrenia Patients: An Exploratory Twelve-Week Randomized, Double-Blind, Placebo-Controlled Study
Source: PLoS One. 2016 Dec 14;11(12):e0168347. doi: 10.1371/journal.pone.0168347 (PMC5156367; doi:10.1371/journal.pone.0168347)
Supplement: S1 File — (PDF) [file pone.0168347.s001.pdf]

臺北醫學大學暨附屬醫院聯合人體研究倫理委員會
TMU-Joint Institutional Review Board

（計畫書封面頁）

試驗計畫名稱：

中文：精神分裂症患者代謝異常之治療: 低劑量 metformin 對服用 clozapine 且有代謝異常之精神分裂患者的效用評估

英文： Treatment of metabolic abnormalities in patients with schizophrenia: Adjunctive low-dose metformin in clozapine-treated patients with schizophrenia and metabolic abnormalities

試驗計畫編號：________________________

試驗計畫版本/日期：__version 1.0 1011230___

（試驗計畫書有任何修正，必須有修正編號及日期。）

試驗委託廠商/機構及聯絡人：(若有)

廠商/機構名稱：
姓名: 單位/職稱：
地址：
辦公室電話: 手機: 傳 真:
電子郵件信箱:

試驗計畫主持人：

姓名: **陳俊興** 單位/職稱：主治醫師（副教授）
地址：台北市興隆路三段 111 號
辦公室電話: 手機: 0970746395 傳 真:
電子郵件信箱: chunhsin57@yahoo.com.tw

試驗計畫協同主持人：(請依需求自行延伸)

姓名: 盧孟良 單位/職稱：主治醫師（副教授）
地址：台北市興隆路三段 111 號
辦公室電話: 手機: 0970746791 傳 真:
電子郵件信箱:

姓名: 單位/職稱：
地址：
辦公室電話: 手機: 傳 真:
電子郵件信箱:

試驗計畫研究護士：

姓名: 單位/職稱：
地址：
辦公室電話: 手機: 傳 真:
電子郵件信箱:

試驗計畫統計人員：

姓名: 單位/職稱：
地址：
辦公室電話: 手機: 傳 真:
電子郵件信箱:

※主持人聲明※

本人負責執行此臨床試驗，依赫爾辛基宣言的精神，藥品優良臨床試驗準則及國內相關法令的規定，來落實執行此計畫。務求確保試驗對象之生命、健康、個人隱私及尊嚴。

計畫主持人姓名：_陳俊興__________

簽章： 陳俊興（簽名） **日期：_101.12.30_________**

摘要

背景：在精神醫學界，許多雙盲-安慰劑控制研究發現 metformin 750-1700 mg/d 可以安全而有效地改善許多代謝異常，例如減輕體重及腹圍，並且降低三酸肝油脂、血糖及胰島素。到目前為止還沒有研究 500 mg/d 的 metformin 是否對代謝異常有效。我們假設在每天 500 mg/d 或 1000 mg/d 的 metformin 皆可有效改善代謝異常，但是這樣的改善在停藥後又回復用藥前的狀態。

目的：本研究的目的有三：第一：篩檢服用 clozapine 患者的代謝狀態（其中部分為過去收案之病人）；第二:評估在代謝異常之精神分裂症患者，低劑量(500 mg/d and 1000 mg/d) metformin 對代謝指標的效應。做為未來長期介入代謝異常患者的先驅研究。第三：追蹤停止介入後代謝指標的改變。

方法：這是一個三階段的研究。第一階段會篩檢服用 clozapine 的精神分裂症患者（其中部分為過去收案的病人）；第二階段是針對在第一階段篩檢中有代謝異常的患者，給予 12 週的雙盲-安慰劑控制研究，隨機分派至三組，分別為 metformin 500 mg/d、 metformin 1000 mg/d，或安慰劑組。第三階段為停止介入後追蹤病人的代謝指標。本研究必先通過機構的人體試驗委員會通過後才開始執行。

第一階段納入標準：1.符合 DSM-IV 精神分裂症或情感性精神分裂症；2.年齡 20-65 歲；3.服用 clozapine 三個月以上。***第一階段排除標準***：無。

第二階段納入標準：在第一階段篩檢有代謝異常者。代謝異常定義為有下列一項或以上者：身體體重指數(BMI)超過 24、腹圍男性超過 90 公分或女性超過 80 公分、三酸肝油脂≥ 150 mg/dL、高密度膽固醇男性小於 40 mg/dL 或女性小於 50 mg/dL; 收縮壓/舒張壓高於或等於 130/ 85 mm Hg 或是目前正在服用高血壓藥物; 空腹血糖高於或等於 100mg/dL。***第二階段排除標準***：有糖尿病病史或目前正在使用糖尿病或高血脂用藥；空腹血糖高於或等於 126 mg/dL 或糖化血色素大於等於 6.5%；女性懷孕；已知對 metformin 過敏者； Creatine>1.4 ng/dl；肝指數異常 (AST 及 ALT 高於正常值二倍者)；慢性心肺功能障礙者;合併酒癮者。

介入過程：在介入期間藥物維持不變動。個案隨機分派到 metformin 500 mg/d、 metformin 1000 mg/day，或安慰劑組。

第二階段的追蹤：身體、精神狀態、及實驗室檢查將在第 2、4、8、12 週定期追踪。

第三階段的追蹤：將在介入結束後的第 4、8、12 週定期追踪代謝指標。

主要成效測量：體重。***次要成效測量***：身體體重指數、代謝症候群的指標：包括腹圍、血壓、空腹三酸肝油脂、高密度膽固醇、及血糖值糖化血色素、胰島素。

計畫書：

Body weight gain and metabolic abnormalities have been one of major side effects in antipsychotic-treated patients with schizophrenia. The prevalence of metabolic syndrome was high in patients with schizophrenia worldwide (1-4). Our study showed that the prevalence of metabolic syndrome was 34.9% and obesity was 35.3% (58%, if including overweight) in patients with schizophrenia in Taiwan(5). Metabolic abnormalities not only result in substantial morbidity and mortality,(6, 7) but also affect functional outcome,(8) self-esteem,(9) and compliance.(10) Therefore, it is important to control metabolic abnormalities in patients with schizophrenia.

Clozapine is the most effective agent for treatment-resistant schizophrenia.(11, 12) However, it is also one of the medications with the greatest propensity to induce body weight gain and metabolic abnormalities among all available antipsychotics.(7, 13) CATIE phase-3 study revealed that patients receiving clozapine increased body weight, and blood levels of glucose, triglyceride (TG), and glycosylated hemoglobin.(14) Other longitudinal studies also found that 36.6% and 43% of clozapine-treated patients were diagnosed with diabetes mellitus (DM) during 5-year and 10-year follow-up, respectively.(15, 16) In addition, the prevalence of metabolic syndrome in clozapine-treated patients was at least 2.5 times of that in the general population.(17) Therefore, it is an important issue to treat clozapine-treated patients with schizophrenia and metabolic abnormalities.

1.1 Lifestyle intervention for metabolic abnormalities

Lifestyle modifications have been proved to successfully prevent the occurrence of metabolic abnormalities (18, 19). Among subjects with impaired glucose tolerance, intensive lifestyle intervention reduced the incidence of DM by 58% (18) and metabolic syndrome (MS) by 41% (19), as compared with placebo. Recent studies suggest that behavioral interventions in patients with schizophrenia may prevent future weight gain, and in some instances promote weight loss (20-23). However, it is difficult to successfully apply behavior and dietary modifications in patients with schizophrenia out of institutions. Hence, effective pharmacological strategies are urgently needed to assist an optimal control of metabolic disturbance in patients with metabolic abnormalities.

1.2 Adjunctive metformin for metabolic abnormalities

In Maayan et al.'s review, among 15 medications, which were tested to attenuate antipsychotic-related weight gain and metabolic abnormalities, metformin is the most effective one. Other effective medications included d-fenfluramine, sibutramine, topiramate, reboxetine (24). In Wu et al's study, they found metformin and lifestyle modification is the most effective way to improve antipsychotic-induced weight gain, but metformin alone was more effective than lifestyle alone to improve body weight gain (25).

Metformin, which is prescribed for patients with non-insulin-dependent diabetes to control blood glucose levels, has been reported to achieve weight loss in several groups of patients characterized by insulin resistance (26, 27). In a longitudinal nearly 3-year follow-up study, compared with placebo group, metformin 1700 mg/day has been shown to reduce the incidence of DM by 31% and MS by 17% in individuals with impaired glucose tolerance (IGT), though less robust than intensive lifestyle intervention did (18, 19).

In psychiatric field, several studies evaluated the effects of metformin on antipsychotics-induced weight gain. Some double-blind, placebo-controlled studies showed that metformin (dose from 750 mg/d to 1700 mg/d) could safely decreased body weight (25, 28-31) and waist circumference (25, 30), and have beneficial effects on lipid profiles (29). Compared to placebo group, metformin could keep the insulin sensitivity unchanged or improve insulin resistance (25, 28, 30).

1.3 Our Past findings in metformin's effects on metabolic features

Our open-labelled study showed that 8-weeks treatment with metformin 1500 mg/d could significantly decrease body weight, fasting levels of glucose, triglyceride, and insulin in olanzapine-treated patients. Half of subjects with metabolic syndrome obtained improvement after metformin trial (32).

Recently, we conducted a 24-week randomized, double-blind, placebo-controlled study of adjunctive metformin for clozapine-treated patients with metabolic abnormalities {Chen, accepted #118}. Our results showed that metformin (titrating from 1000 to 1500 mg/d at week 1 to week 2) had beneficial effects on some metabolic indices among clozapine-treated schizophrenic patients. These effects appeared at as early as 2nd to 4th weeks (see Fig 1).

Figure 1. Trends of metabolic index changes in the metformin and the placebo groups
Abbreviations: BMI = body mass index, BW = body weight, DBP = diastolic blood pressure, FPG = fasting plasma glucose, HDL-C = high density lipoprotein cholesterol, HOMA-IR = homeostasis model assessment-insulin resistance, SBP = systolic blood pressure, TG = triglyceride, WC = waist circumference.

BW

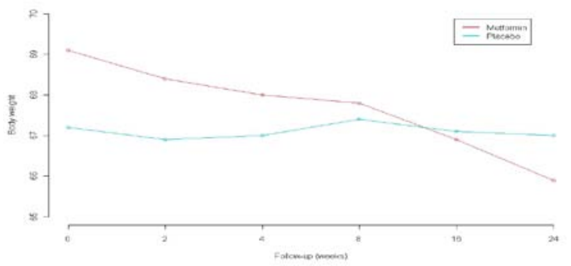

BMI

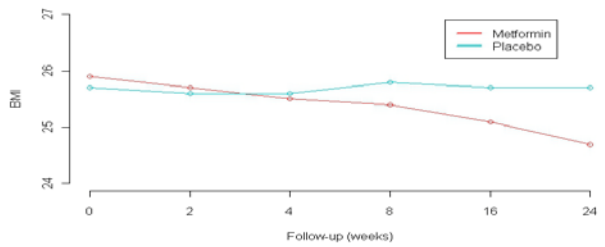

WC

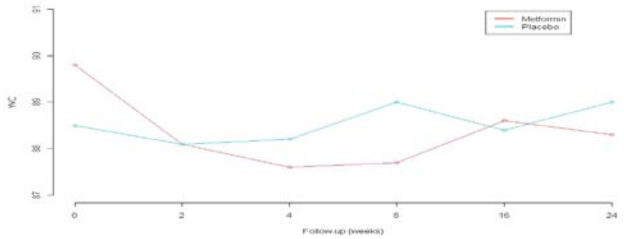

SBP

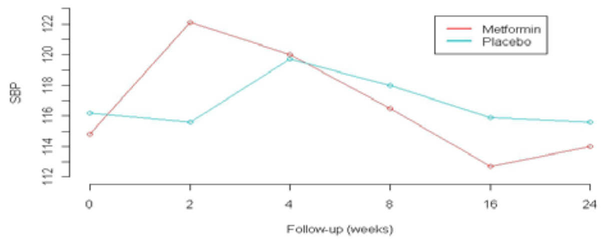

DBP

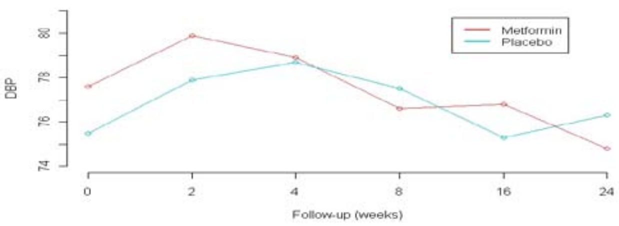

TG

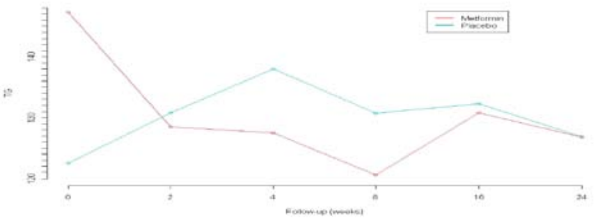

FPG

HDL-C

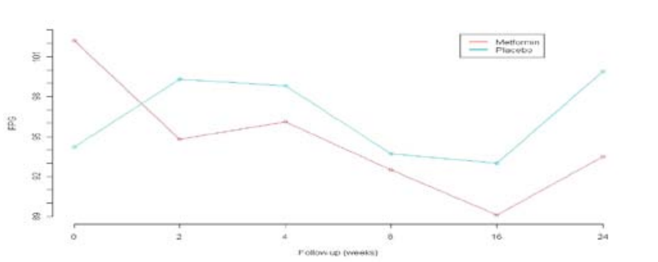

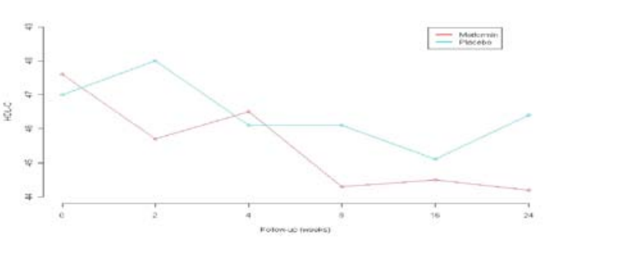

Insulin

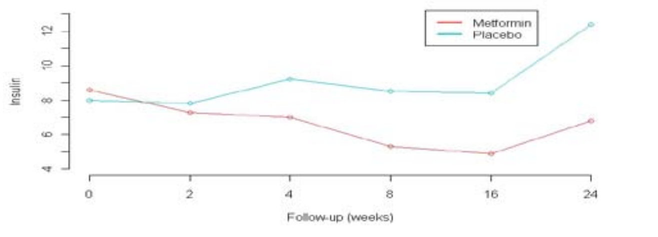

HOMA-IR

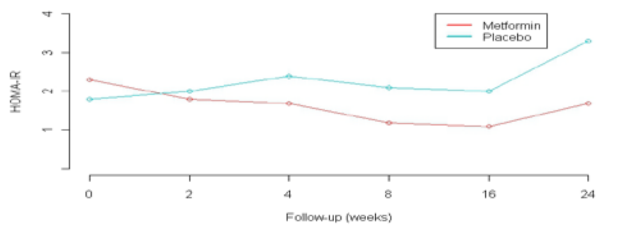

1.4 Dose-effect of metformin on metabolic features

From past studies using Chinese Han as subjects, the effective metformin dose was from 750 mg/d (25, 30) to 1000 mg/d (31, 33). Though no severe adverse effect was reported in our previous trials with 1500 mg/d of metformin (32, 34), the determination of minimal effective dose of metformin is important, if we would like to long-termly use metformin to prevent or reverse metabolic abnormalities in patients with schizophrenia in clinical practice. From our previous study, we used 1000 mg/d in the first week and then 1500 mg/d in the second week. We found some metabolic features, such as waist circumference, TG, FPG, and insulin levels, have significant changes at week-2 follow-up. (Fig 2) There was also a trend (not significant) of body weight decrease at week-2. (34) Therefore, we hypothesize 1000 mg/d of metformin would have beneficial effects on metabolic features in clozapine-treated patients who had metabolic abnormalities.

One recent study compared the effect of metformin 1500-1700 mg/d and 1000 mg/d on endocrine and metabolic features in patients with polycystic ovary syndrome (PCOS). They found that metformin exerted an overall positive effect on endocrine and metabolic features of PCOS. The degree of these effects was independent of the administered dosage in every range of BMI. (35) It suggests that low dose of metformin might have beneficial effects on metabolic features. Although there were several clinical trials proved that 750-1700 mg of metformin could have positive effects on metabolic features in patients with schizophrenia, (25, 28-31) until now, no study compared the effects of different doses, especially as low as 500 mg/d, of metformin on metabolic features in patients with schizophrenia and preexisting metabolic adversities.

1.5 Discontinuation of metformin on metabolic features

Though many studies have supported the efficacy of adjunctive metformin on antipsychotic-treated patients, only our previous trial followed patients' body weight after discontinuation of metformin. Our previous trial followed patients' body weight after they stop taking metformin at least 24 weeks. We found that body weight returned to baseline at 6 months after stopping metformin (see Fig 2). (34) Though we found that 28.7% patients, who fulfilled metabolic syndrome at baseline, did not meet the criteria of metabolic syndrome after 24-week metformin intervention, we did not know whether their status of metabolic syndrome return to baseline after stopping metformin. We hypothesize that not only body weight, but metabolic features, would return to baseline after patients stop taking metformin.

Figure 2. Trends of body weight (mean) changes in metformin and placebo groups in baseline, 24-week follow up and 24-week follow-up without medication

1.6 Aims and hypotheses

Our study aims are:

1. to follow previously recruited patients' metabolic features and screen more clozapine-treated patients with schizophrenia.
2. to compare the effectiveness of 500 mg/d, 1000mg/d of metformin, and placebo on metabolic features in clozapine-treated schizophrenia patients with metabolic abnormalities, which will be defined in the following section.
3. to monitor the metabolic profile changes after stopping metformin.

We hypothesize that lower dose (500 mg/d or 1000 mg/d) of metformin would have beneficial effects on metabolic features and cytokines in patients with metabolic abnormalities, and the beneficial effects disappear after stopping metformin.

（三）研究方法、進行步驟及執行進度。

This will be a three-phase study. The first phase is to screen clozapine-treated patients with schizophrenia in outpatient clinics and inpatients in rehabilitation wards. The second phase is a 12-week randomized, double-blind, placebo-controlled study of adjunctive metformin (500 or 1000 mg/d) or placebo for clozapine -treated patients with metabolic abnormalities. The third phase is to follow patients' metabolic features after they stop taking metformin at week 4, 8, and 12. The study will be approved by the Institutional Review Board of participated institutions before recruiting patients.

2.1 Subjects

1. First phase screening:

In the first screen phase, the inclusion criteria are:

1). fulfill DSM-IV criteria of schizophrenia or schizoaffective disorder;

2). age 20 to 65 years;

3). receive clozapine for at least 3 months;

We will invite our previously recruited patients first and then screen more clozapine-treated patients in outpatient clinics and rehabilitation ward.

2. Second phase intervention

Those who meet the following inclusion and exclusion criteria in the first phase screen will be invited to participate in the second phase intervention study.

The inclusion criteria are: have metabolic abnormalities.

The metabolic abnormalities are defined as at least one of the following conditions:

1. overweight or obese (BMI ≥ 24)
2. abdominal obesity (waist circumference > 90 cm, in men and > 80 cm, in women)
3. fasting hypertriglyceridemia, (≥ 150 mg/dL);
4. low fasting HDL levels (< 40 mg/dL in men and < 50 mg/dL in women);
5. high blood pressure (≥ 130/ ≥ 85 mm Hg or current treatment with antihypertensive medications).
6. FPG levels ≥ 100 mg/dL

The exclusion criteria are:

1. history of DM;
2. current use of hypoglycemic or hypolipidemic agents;
3. FPG levels ≥ 126 mg/dL or HbA1c ≥ 6.5%;
4. women who are pregnant;

5. known allergy
6. Creatine >1.4 ng/dl;
7. abnormal liver function test (AST and ALT higher than 70)
8. history of chronic cardiopulmonary insufficiency.
9. alcohol dependence

2.2 Evaluation and measurements

Recruited patients' data will be collected from clinical interview and medical records, which include demographic characteristics and clinical information, such as diagnosis, age at onset of schizophrenia or schizoaffective disorder, initiation and dose of current antipsychotics and other co-medications.

All the following assessments will be conducted by trained research assistants or psychiatrists, and collaborative laboratory. Assessments include physical examination, psychiatric and side effect assessments, and laboratory assay.

(1) Physical examination:
- a. Height,
- b. body weight,
- c. waist circumference (WC),
- d. sitting blood pressure (BP)
- e. general medical examination

(2) Psychiatric and side effect assessments
- a. PANSS (36)
- b. Clinical Global Impression-Severity of Illness scale (CGI-S).
- c. The Udvalg for Kliniske Undersogelser (UKU) Side Effect Rating Scale (37) will be used for monitoring both extrapyramidal symptoms and other side-effect profiles.

(3) Laboratory assay

Overnight fasting blood will be collected for the following assays:
- a. Blood routine, including RBC, Hb, Hct, WBC etc.
- b. General biochemistry, such as liver function test, renal function test, and electrolytes
- c. Metabolic-related examinations, including fasting plasma glucose (FPG), TG, HDL-C, insulin, and HbA1c

Urine pregnancy test will be checked for potential pregnant women.

2.3 Intervention

The antipsychotic dosages will be maintained unchanged during the study period. If those patients whose current antipsychotics (clozapine) are switched to another kind of antipsychotics due to medical or psychiatric considerations (not include dosing adjustment), they are regarded as withdrawal from the study.

Recommendations for healthy food and exercise to control body weight will be provided at the beginning of the study, but not during the follow-up visits.

2.3.1 Blinding procedure:

The randomization allocation will be conducted by a research assistant who is blind to the status of participants. Then eligible patients will be randomized to metformin either 500 mg/d, 1000 mg/d, or identical-appearance placebo groups. Patients, caregivers, and investigators are all

masked to the assignment. Placebo will be identical-appearing starch tablet and will be produced by GMP pharmaceutical manufacturer.

2.3.2 Dosing strategy

1. Metformin 500 mg/d or placebo (1# in the morning) during the first week of intervention will be provided.

2. Then medications will be titrated to 1# (metformin 500 mg for metformin 500 mg/d and 1000mg/d groups, or placebo) in the morning and 1# (500 mg for metformin 1000 mg/d group, or placebo for 500 mg/d and placebo groups) in the afternoon at the second week to reach the corresponding target dose, unless subjects can not tolerate side effects induced by metformin.

3. For those who can not tolerable target metformin dose at week 4, the tolerable metformin dose at week 4 will be kept during the following intervention period.

2.4 Follow-up assessments

Physical examination, psychiatric assessment, and laboratory assay will be repeated at week-2, 4, 8, and 12. The laboratory tests will be performed in the morning after an overnight fasting. We followed patients' metabolic features at week 4, 8, and 12 after they stop taking medications.

2.5 Outcomes measurement

Primary outcome is body weight change.

Secondary outcomes include changes of other metabolic profiles' level, such as waist circumference, BP, TG, HDL-C, FPG, insulin and HOMA-IR.

2.6 Sample size estimation

Our sample size estimation is determined by our primary outcome measurement, i.e. body weight.

From our preliminary data showed that the mean body weight decrease was 2.2 Kg after 16-week and 3.2 Kg after 24-week of 1500 mg/d of metformin intervention. Another Chinese Han study also showed a mean 3.2 Kg of body weight decrease in 12-week 750 mg/d of metformin treatment (25). Therefore, we set the difference of body weight change is 2 Kg in metformin group (either 500 or 1000 mg/d) and 0 Kg in placebo group; a power of 80% and a type I error of 5%. The standard deviation is 2 Kg. The minimal size for each group will be 16 subjects. Considering potential drop-out, we will recruit 20 patients in each group in our current study.

2.7 Statistical analysis

We adopt last observation carried forward (LOCF). The data of patients who stay in the second phase at least 4 weeks were included into analyses.

1. Descriptive statistics are represented as mean ± SD.

2. Subgroups were compared using t-test for continuous variables and chi-square test for categorical variables

3. For all outcome measures, including body weight, BMI, and metabolic features, we analyzed the changes from baseline by using a repeated-measures analysis of variance, controlling for baseline. Least-significant-difference procedure was used for post hoc comparisons among metabolic profiles at different time points.

4. A p-value of less than 0.05 was considered to have statistical significance.

3. Anticipated results and future applications

We anticipate that metformin 500 mg/d and 1000 mg/d can have beneficial effects on patients with body weight and metabolic features. These beneficial effects would disappear after stop taking metformin.

Our long-term goal is to use metformin in psychiatric patients, who have metabolic abnormalities. We think that adjunctive metformin is an inexpensive and easy way to control metabolic abnormalities in those subjects, for whom intensive lifestyle change is difficult.

2.預計可能遭遇之困難及解決途徑。

a. 可能遭遇的困難包括收案進度落後,如果在同一醫院案困難,將會找其他醫院合作,共同收案。

b. 如果是多中心合作,將又面臨評估個案一致性問題,所以將一家一家醫院收案,以同一位助理做評估,如果有其他人員同時進行評估,則事前先進行訓練，讓評估的一致性提高。實驗方面將統一由同一實驗室執行。

References

1. Bobes J, Arango C, Aranda P, Carmena R, Garcia-Garcia M, Rejas J. Cardiovascular and metabolic risk in outpatients with schizophrenia treated with antipsychotics: results of the CLAMORS Study. Schizophr Res. 2007;90(1-3):162-73.
2. De Hert MA, van Winkel R, Van Eyck D, Hanssens L, Wampers M, Scheen A, et al. Prevalence of the metabolic syndrome in patients with schizophrenia treated with antipsychotic medication. Schizophr Res. 2006;83(1):87-93.
3. Hagg S, Lindblom Y, Mjorndal T, Adolfsson R. High prevalence of the metabolic syndrome among a Swedish cohort of patients with schizophrenia. Int Clin Psychopharmacol. 2006;21(2):93-8.
4. McEvoy JP, Meyer JM, Goff DC, Nasrallah HA, Davis SM, Sullivan L, et al. Prevalence of the metabolic syndrome in patients with schizophrenia: baseline results from the Clinical Antipsychotic Trials of Intervention Effectiveness (CATIE) schizophrenia trial and comparison with national estimates from NHANES III. Schizophr Res. 2005;80(1):19-32.
5. Huang MC, Lu ML, Tsai CJ, Chen PY, Chiu CC, Jian DL, et al. Prevalence of metabolic syndrome among patients with schizophrenia or schizoaffective disorder in Taiwan. Acta psychiatrica Scandinavica. 2009;120(4):274-80.
6. Hennekens CH, Hennekens AR, Hollar D, Casey DE. Schizophrenia and increased risks of cardiovascular disease. Am Heart J. 2005;150(6):1115-21.
7. Newcomer JW. Second-generation (atypical) antipsychotics and metabolic effects: a comprehensive literature review. CNS Drugs. 2005;19 Suppl 1:1-93.
8. Lyketsos CG, Dunn G, Kaminsky MJ, Breakey WR. Medical comorbidity in psychiatric inpatients: relation to clinical outcomes and hospital length of stay. Psychosomatics. 2002;43(1):24-30.
9. De Hert M, Peuskens B, van Winkel R, Kalnicka D, Hanssens L, Van Eyck D, et al. Body weight and self-esteem in patients with schizophrenia evaluated with B-WISE. Schizophr Res. 2006;88(1-3):222-6.

10. Weiden PJ, Mackell JA, McDonnell DD. Obesity as a risk factor for antipsychotic noncompliance. Schizophr Res. 2004;66(1):51-7.
11. Kane J, Honigfeld G, Singer J, Meltzer H. Clozapine for the treatment-resistant schizophrenic. A double-blind comparison with chlorpromazine. Arch Gen Psychiatry. 1988;45(9):789-96.
12. Tandon R, Belmaker RH, Gattaz WF, Lopez-Ibor JJ, Jr., Okasha A, Singh B, et al. World Psychiatric Association Pharmacopsychiatry Section statement on comparative effectiveness of antipsychotics in the treatment of schizophrenia. Schizophr Res. 2008;100(1-3):20-38.
13. Allison DB, Mentore JL, Heo M, Chandler LP, Cappelleri JC, Infante MC, et al. Antipsychotic-induced weight gain: a comprehensive research synthesis. Am J Psychiatry. 1999;156(11):1686-96.
14. Stroup TS, Lieberman JA, McEvoy JP, Davis SM, Swartz MS, Keefe RS, et al. Results of phase 3 of the CATIE schizophrenia trial. Schizophr Res. 2009;107(1):1-12.
15. Henderson DC, Cagliero E, Gray C, Nasrallah RA, Hayden DL, Schoenfeld DA, et al. Clozapine, diabetes mellitus, weight gain, and lipid abnormalities: A five-year naturalistic study. Am J Psychiatry. 2000;157(6):975-81.
16. Henderson DC, Nguyen DD, Copeland PM, Hayden DL, Borba CP, Louie PM, et al. Clozapine, diabetes mellitus, hyperlipidemia, and cardiovascular risks and mortality: results of a 10-year naturalistic study. J Clin Psychiatry. 2005;66(9):1116-21.
17. Lamberti JS, Olson D, Crilly JF, Olivares T, Williams GC, Tu X, et al. Prevalence of the metabolic syndrome among patients receiving clozapine. Am J Psychiatry. 2006;163(7):1273-6.
18. Knowler WC, Barrett-Connor E, Fowler SE, Hamman RF, Lachin JM, Walker EA, et al. Reduction in the incidence of type 2 diabetes with lifestyle intervention or metformin. N Engl J Med. 2002;346(6):393-403.
19. Orchard TJ, Temprosa M, Goldberg R, Haffner S, Ratner R, Marcovina S, et al. The effect of metformin and intensive lifestyle intervention on the metabolic syndrome: the Diabetes Prevention Program randomized trial. Ann Intern Med. 2005;142(8):611-9.
20. Loh C, Meyer JM, Leckband SG. A comprehensive review of behavioral interventions for weight management in schizophrenia. Ann Clin Psychiatry. 2006;18(1):23-31.
21. McKibbin CL, Patterson TL, Norman G, Patrick K, Jin H, Roesch S, et al. A lifestyle intervention for older schizophrenia patients with diabetes mellitus: a randomized controlled trial. Schizophr Res. 2006;86(1-3):36-44.
22. Bushe C, Haddad P, Peveler R, Pendlebury J. The role of lifestyle interventions and weight management in schizophrenia. J Psychopharmacol. 2005;19(6 Suppl):28-35.
23. Wu MK, Wang CK, Bai YM, Huang CY, Lee SD. Outcomes of obese, clozapine-treated inpatients with schizophrenia placed on a six-month diet and physical activity program. Psychiatr Serv. 2007;58(4):544-50.
24. Maayan L, Vakhrusheva J, Correll CU. Effectiveness of medications used to attenuate antipsychotic-related weight gain and metabolic abnormalities: a systematic review and meta-analysis. Neuropsychopharmacology. 2010;35(7):1520-30.
25. Wu RR, Zhao JP, Jin H, Shao P, Fang MS, Guo XF, et al. Lifestyle intervention and metformin for treatment of antipsychotic-induced weight gain: a randomized controlled trial. JAMA. 2008;299(2):185-93.
26. Glueck CJ, Fontaine RN, Wang P, Subbiah MT, Weber K, Illig E, et al. Metformin reduces weight, centripetal obesity, insulin, leptin, and low-density lipoprotein cholesterol in

nondiabetic, morbidly obese subjects with body mass index greater than 30. Metabolism. 2001;50(7):856-61.
27. Velazquez EM, Mendoza S, Hamer T, Sosa F, Glueck CJ. Metformin therapy in polycystic ovary syndrome reduces hyperinsulinemia, insulin resistance, hyperandrogenemia, and systolic blood pressure, while facilitating normal menses and pregnancy. Metabolism. 1994;43(5):647-54.
28. Baptista T, Rangel N, Fernandez V, Carrizo E, El Fakih Y, Uzcategui E, et al. Metformin as an adjunctive treatment to control body weight and metabolic dysfunction during olanzapine administration: a multicentric, double-blind, placebo-controlled trial. Schizophr Res. 2007;93(1-3):99-108.
29. Carrizo E, Fernandez V, Connell L, Sandia I, Prieto D, Mogollon J, et al. Extended release metformin for metabolic control assistance during prolonged clozapine administration: a 14 week, double-blind, parallel group, placebo-controlled study. Schizophr Res. 2009;113(1):19-26.
30. Wu RR, Zhao JP, Guo XF, He YQ, Fang MS, Guo WB, et al. Metformin addition attenuates olanzapine-induced weight gain in drug-naive first-episode schizophrenia patients: a double-blind, placebo-controlled study. Am J Psychiatry. 2008;165(3):352-8.
31. Wang M, Tong JH, Zhu G, Liang GM, Yan HF, Wang XZ. Metformin for treatment of antipsychotic-induced weight gain: A randomized, placebo-controlled study. Schizophr Res. 2012;138(1):54-7.
32. Chen CH, Chiu CC, Huang MC, Wu TH, Liu HC, Lu ML. Metformin for metabolic dysregulation in schizophrenic patients treated with olanzapine. Prog Neuropsychopharmacol Biol Psychiatry. 2008;32(4):925-31.
33. Wu RR, Jin H, Gao K, Twamley EW, Ou JJ, Shao P, et al. Metformin for treatment of antipsychotic-induced amenorrhea and weight gain in women with first-episode schizophrenia: a double-blind, randomized, placebo-controlled study. Am J Psychiatry. 2012;169(8):813-21.
34. Chen CHH, M.C.; Kao, C.F.; Lin, S.K.; Kuo, P.H.; Chiu, C.C.; Lu, M.L. Effects of Adjunctive Metformin on Metabolic Traits in Non-diabetic Clozapine-treated Patients with Schizophrenia: A 24-week, Randomized, Double-blind, Placebo-controlled Study and Its Discontinuation Effect on Body Weight. J Clin Psychiatry. accepted.
35. Fulghesu AM, Romualdi D, Di Florio C, Sanna S, Tagliaferri V, Gambineri A, et al. Is there a dose-response relationship of metformin treatment in patients with polycystic ovary syndrome? Results from a multicentric study. Hum Reprod. 2012;27(10):3057-66.
36. Kay SR, Fiszbein A, Opler LA. The positive and negative syndrome scale (PANSS) for schizophrenia. Schizophrenia bulletin. 1987;13(2):261-76.
37. Lingjaerde O, Ahlfors UG, Bech P, Dencker SJ, Elgen K. The UKU side effect rating scale. A new comprehensive rating scale for psychotropic drugs and a cross-sectional study of side effects in neuroleptic-treated patients. Acta psychiatrica Scandinavica Supplementum. 1987;334:1-100.

赫爾辛基宣言（Declaration of Helsinki）
2000 年中文版

甲.引言

1. 世界醫學會制定赫爾辛基宣言，作為醫師及醫學研究人員在人體試驗時之倫理指導原則。而所謂人體試驗之對象即包涵任何可辨識之人體組織或資料。
2. 醫師之職責在促進及維護人類之健康，其專業知識及良知應奉獻於此一使命。
3. 世界醫學會之日內瓦宣言（Declaration of Geneva）中，規範醫師必須以“病患之福祉為首要之考量”，而國際醫療倫理規章（International Code of Medical Ethics）亦宣示“在實施任何可能危及病患身心之醫療措施時，醫師應以病患之福祉為唯一之考慮。”
4. 醫學之進步奠基於科學研究，而此研究終究必須有部份仰賴以人為受試驗者。
5. 在進行有關人體試驗之醫學研究時，應將受試驗者之利益置於科學及社會利益之上。
6. 進行人體醫學實驗之首要目的，在於改進各種預防、診斷及治療之方法，及增進對於疾病成因之瞭解。對於目前已知最有效之預防、診斷及治療之方法，也應不斷地以研究來檢證其效果，效率，可行性，及品質。
7. 在當前的醫療行為及醫學研究中，大多數的預防、診斷及治療程序都涉及一定的危險與醫療責任。
8. 醫學研究之倫理標準，應以尊重生命，維護人類之健康及利益為依歸。對於較易受傷之受測族群必須特別加以保護。經濟弱勢及醫療資源匱乏之族群的特別需求也應加以關注。對於無法自行同意或拒絕研究的人、對於可能在脅迫下行使同意的人、對於那些無法因研究而親身受惠的人、及那些同時接受研究和醫療照護的人，也應特別關注。
9. 試驗主持人應注意該國與人體試驗有關之倫理、法律、及主管機關相關規定及適用的國際法規。任何國家之倫理、法律、條例之制定，皆不應減損或忽視本宣言對受試驗者所宣示之保障。

乙.醫學研究之基本原則

10. 醫學研究中，醫師之職責是在於保障受試驗者之生命、健康、個人隱私及尊嚴。
11. 任何涉及人體試驗之醫學研究，必須依循普遍接受之科學原則，並奠基於對科學文獻之徹底瞭解，相關資訊之掌握，及適當的研究數據及動物實驗。
12. 對於可能影響環境之研究都必須謹慎進行，而實驗動物之福祉也應予以尊重。
13. 在實驗計劃中，有關人體試驗的每一個實驗步驟，皆應清楚陳述其實驗之設計與執行。此試驗計畫書必須交由一特別任命之倫理審查委員會，加以考查、評判及指導，如果適當，才予以核准。此倫理審查委員會，必須獨立於研究者、資助者、或任何其他不當影響力之外。此獨立委員會應遵守該研究實驗所在國的法律及規定。委員會應有權監測進行中的試驗。研究人員有責任向委員會提供實驗監測資訊，特別是任何嚴重不良事件。研究人員應向委員會提供資訊以供審查，包括其研究經費、試驗委託者、所屬機構，及其潛在的利益衝突，和受試驗者參與實驗之誘因。
14. 試驗計畫書需檢附相關倫理考量的聲明，並得符合本宣言所揭櫫之原則。
15. 凡涉及人體試驗的醫學研究，皆須由受過科學訓練的合格人員執行，並由合格臨床醫療人員的監督下進行。對於人體試驗所產生的責任歸屬，皆由合格的醫療人員負責；即使事前已徵得該受試驗者之同意，該受試驗者亦不需負任何責任。
16. 任何有關人體試驗的醫療研究計劃，事前須審慎評估可能的風險、責任、以及對受試驗者或其他人的可能益處。此種評估亦應涵括參與研究的健康志願者。所有研究的設計皆應開放供大眾取得。
17. 除非醫師已充份評估可能產生的風險，並自信能充分地掌控實驗，否則應避免從事有關人體試驗的研究計劃。一旦發現實驗的風險高過其潛在的利益；或已可得到正面或有益之結論時，醫師即應停止其研究計劃。
18. 唯有在研究目的之重要性大於受試驗者可能身受的風險時，有關人體試驗的醫學研究才可以進行。當該受試驗者為健康的志願者時，尤需重視此原則。
19. 唯有被研究的族群可能從醫學研究成果中獲益時，此醫學研究才有其執行之價值。
20. 受試驗者必須是志願參加，並充份瞭解研究內容，才得以參與該項研究計劃。
21. 受試驗者保護其本人身心健全與完整性的權利必須加以尊重。研究人員應採取一切之預防措施，尊重受試驗者之個人隱私，維護其個人資料的私密，並將此研究對其身心健全及人格造成之傷害降到最低。

22. 在任何人體試驗中，每一個可能的受試驗者，必須被告知該研究的目的、方法、經費來源、任何可能的利益衝突、研究人員所屬機構、該研究可預見的益處，及可能伴隨的危險與不適。受試驗者也應被告知其擁有的權利，包括可拒絕參與研究，或可隨時撤回同意而不受報復。在確知受試驗者已充分瞭解以上訊息後，醫師應取得受試驗者於自由意志下簽署之受試同意書，此受試同意書以書面行之為佳。若受試同意書無法以書面方式行之，則非書面之同意必須經過正式地紀錄與見證。
23. 醫師在取得受試同意書時，應特別注意受試驗者是否對醫師有依賴關係，或受試驗者是否在脅迫下行使同意。在此情況下，此受試同意書應由一位充分瞭解全盤研究，但沒有參與研究，並完全與彼無關係的醫師取得。
24. 若受試驗者無法律上之行為能力，或生理或心智上無同意能力，或無法律上行為能力之未成年者，研究人員必須取得符合適用法令之法定代理人受試同意書。除非研究本身有其促進上述族群健康之必要性，而研究又無法於法律上具行為能力之人員上施行，否則此研究不應包涵此類族群。
25. 當一個被視為無法律行為能力之受試驗者，例如未成年之孩童，對參與研究的決定有表達同意之能力時，研究人員除了應取得該受試驗者之同意外，亦必須取得其法定代理人之同意。
26. 當無法從個人取得同意，包括代理人同意或預先同意時，此項對於個人之研究不應進行；除非阻止其簽署受試同意書的個人特殊身心狀況，正是此受試驗者族群的必然特徵。對於此種在無法簽署受試同意書之受試驗者上的研究，研究人員應於試驗計畫書中，陳述其研究之具體原因，以供審查委員會之考量而核准。試驗計畫書中應表明，會儘速從本人，或合法授權之代理人處，取得繼續參與此研究之同意。
27. 作者及出版者皆負道德責任。研究人員在發表研究成果時，即有責任保持其結果的正確性。正面與負面的研究結果都應發表，或可公開取得。研究人員之經費來源，其所屬組織，或研究中任何可能之利益爭議皆應公佈於出版之中。凡不合乎此宣言之原則的實驗報告，皆不該被接受發表。

丙.兼顧醫療照護的醫學研究之附加原則

28. 醫師可以結合醫學研究與醫療照護，但此情況僅止於此研究有潛在的預防、診斷或治療的價值。當醫學研究結合醫療照護時，另有額外的準則來保護這些同為病患和研究對象的人。
29. 一個新醫療方法的益處、危險性、責任、及其效果，應與目前已知最佳的預防、診斷與治療方法對照檢驗。而對於尚無有效預防、診斷與治療方式之研究，不排除使用安慰劑或不予治療來檢驗其療效。
30. 研究結束後，每一個參與研究的病患，都應得到保證其可以接受經此研究證實為最佳的預防、診斷和治療的方法。
31. 醫師應全盤告知病患，那些方面的醫療照護與研究有關。病患的拒絕參與研究，絕對不應影響醫病關係。
32. 在治療病患的過程中，若無有效的預防，診斷和治療的方法，醫師在取得病患之受試同意書後，得以自由採用其判斷下有希望挽救生命，重建健康或減輕痛苦的任何未經證實或新的預防，診斷及治療方法。這些方法，在可能的情況下，應被當作研究的目標，來評估其安全性及有效性。在各種情況下，應將新的消息資訊紀錄，適當時並發表，並應遵守此份宣言的其他相關準則。
